# Transgenic overexpression of VEGF-C induces weight gain and insulin resistance in mice

**DOI:** 10.1038/srep31566

**Published:** 2016-08-11

**Authors:** Sinem Karaman, Maija Hollmén, Sun-Young Yoon, H. Furkan Alkan, Kari Alitalo, Christian Wolfrum, Michael Detmar

**Affiliations:** 1 Institute of Pharmaceutical Sciences, Swiss Federal Institute of Technology, ETH Zurich, 8093 Zurich, Switzerland; 2 Wihuri Research Institute and Translational Cancer Biology Program, Biomedicum Helsinki, University of Helsinki, Finland; 3 Institute of Food, Nutrition and Health, Swiss Federal Institute of Technology, ETH Zurich, 8603 Schwerzenbach, Switzerland

## Abstract

Obesity comprises great risks for human health, contributing to the development of other diseases such as metabolic syndrome, type 2 diabetes and cardiovascular disease. Previously, obese patients were found to have elevated serum levels of VEGF-C, which correlated with worsening of lipid parameters. We recently identified that neutralization of VEGF-C and -D in the subcutaneous adipose tissue during the development of obesity improves metabolic parameters and insulin sensitivity in mice. To test the hypothesis that VEGF-C plays a role in the promotion of the metabolic disease, we used K14-VEGF-C mice that overexpress human VEGF-C under control of the keratin-14 promoter in the skin and monitored metabolic parameters over time. K14-VEGF-C mice had high levels of VEGF-C in the subcutaneous adipose tissue and gained more weight than wildtype littermates, became insulin resistant and had increased ectopic lipid accumulation at 20 weeks of age on regular mouse chow. The metabolic differences persisted under high-fat diet induced obesity. These results indicate that elevated VEGF-C levels contribute to metabolic deterioration and the development of insulin resistance, and that blockade of VEGF-C in obesity represents a suitable approach to alleviate the development of insulin resistance.

The rising incidence of overweight and obesity worldwide has become one of the principal global public health issues of the 21^st^ century^1^. Among other serious health implications, obesity is considered the most frequent cause of insulin resistance, which is a key player in the development of the metabolic syndrome – a multifactorial disease that comprises diverse metabolic abnormalities^2^. In-depth studies in this field indicate that obesity-induced metabolic inflammation in adipose tissue is one of the main contributors to the development of insulin resistance, creating a new niche for metabolic research, i.e. “immunometabolism”^3^,^4^. Adipose tissue is made up of adipocytes as well as hematopoietic, endothelial and other mesenchymal cells, collectively referred to as the stromal-vascular fraction (SVF)^3^. Chronic low-grade inflammation and an increase in inflammatory cytokine levels in the adipose tissue during obesity play a critical role in attracting proinflammatory cells –especially macrophages– into the SVF compartment^5^,^6^.

Vascular endothelial growth factors (VEGFs) are key regulators of angiogenesis and lymphangiogenesis, with diverse roles in pathological conditions such as inflammation and tumor metastasis^7^,^8^. Serum concentrations of different VEGFs seem to be altered during the development of obesity. For instance, levels of VEGF-C, the major lymphangiogenic growth factor, are elevated in the sera of obese patients^9^,^10^,^11^, which correlates with metabolic and lipid parameters^9^. *Vegfc* mRNA levels are also increased in the adipose tissues of both genetic and diet-induced obesity in mice, suggesting that adipose tissue might be one of the sources of elevated VEGF-C levels in obesity^12^. Importantly, VEGF-C has been found to be a chemoattractant factor for macrophages^12^,^13^ and proinflammatory, M1-like macrophages upregulate its cognate receptor VEGFR-3^12^,^14^. Recently, we found that both transgenic and antibody-mediated blockade of VEGF-C/D signaling in subcutaneous adipose tissue of mice reduced inflammatory macrophage infiltration, inflammatory cytokine expression and hepatic steatosis, and also improved insulin sensitivity in both diet-induced and genetic obesity^12^. Since VEGF-D knock-out mice show no metabolic phenotype linked to adipose tissue development when subjected to a high-fat diet^15^, we hypothesize that the blockade of VEGF-C, rather than VEGF-D is responsible for the metabolic improvement observed in the aforementioned study. Hence, in the present study, we investigated the metabolic consequences of transgenic overexpression of VEGF-C, using a transgenic mouse model with expression of human VEGF-C in the skin under control of the keratin-14 promoter^16^. Our results reveal that elevated VEGF-C levels in subcutaneous adipose tissue contribute to the development of adipocyte hypertrophy, ectopic lipid accumulation and insulin resistance.

## Results

### VEGF-C is upregulated in the subcutaneous adipose tissue of obese mice

**Figure 1:**
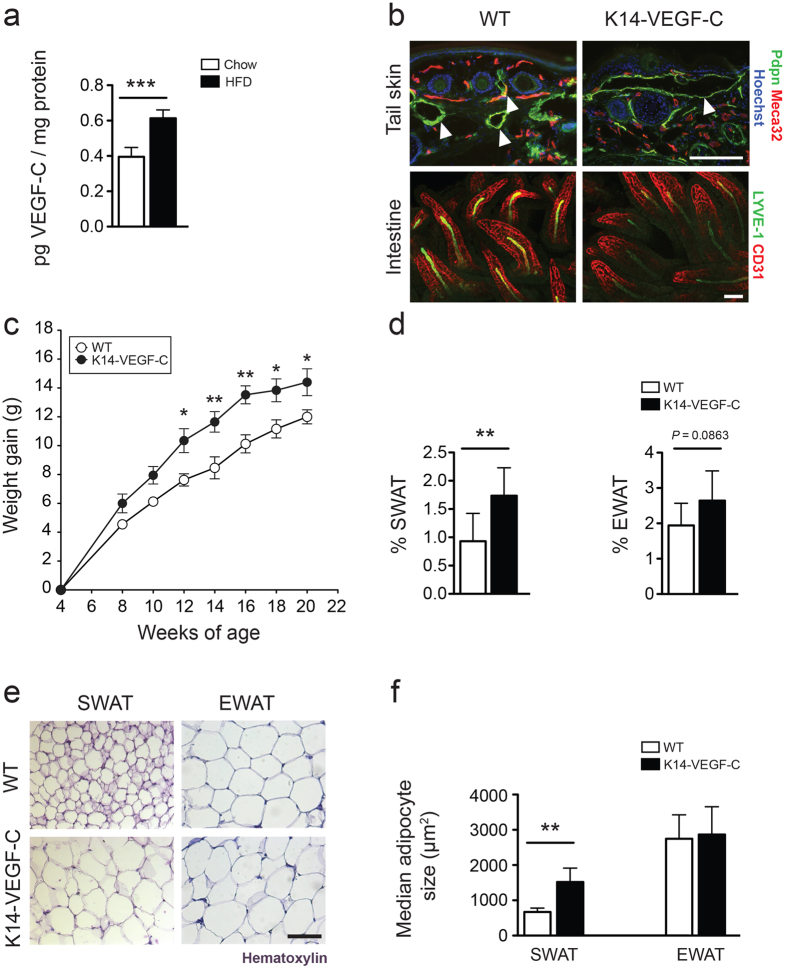
1 Increased weight gain and adipose tissue accumulation in K14-VEGF-C mice. (**a**) ELISA analysis revealed increased murine VEGF-C levels in SWAT of obese mice that were kept under HFD for 20 weeks. (**b**) Immunofluorescence analysis of tail skin showing enlarged lymphatic vessels (podoplanin, Pdpn, green) in K14-VEGF-C mice, while whole-mount immunostains of intestinal villi showed no differences in lacteal (LYVE-1, green) or blood vessel (CD31, red) structure between WT and K14-VEGF-C mice. (**c**) K14-VEGF-C mice gained more weight than WT littermates (*n* = 8 per group, mean ± SEM is shown, *p < 0.05 and **p < 0.01 compared to WT control at the same time point with a two-tailed Student’s *t*-test; effect of genotype: *p* < 0.0001 with a two-way ANOVA), and (**d**) had increased % of SWAT, but not EWAT. (**e,f**) K14-VEGF-C mice had larger adipocytes in SWAT. Scale bars = 100 μm. **p < 0.01, ***p < 0.001, two-tailed Student’s *t*-test. Data are mean ± SD.

We previously found increased *Vegfc* mRNA levels in subcutaneous adipocytes isolated from obese mice^12^. To investigate whether VEGF-C is also increased at the protein level in murine adipose tissue in obesity, we performed ELISA to measure VEGF-C levels in the subcutaneous white adipose tissue (SWAT). We found significantly elevated VEGF-C protein levels in the SWAT of obese mice after 20 weeks on HFD compared to lean mice (lean: 0.40 ± 0.05, *n* = 4; obese: 0.61 ± 0.05 pg VEGF-C/mg protein, *n* = 4; *p* = 0.0009), suggesting that SWAT may be one of the sources of elevated VEGF-C levels in obesity (Fig. 1a). Because transgenic and antibody-mediated blockade of VEGF-C and -D signaling protected mice from obesity-associated metabolic deterioration in our previous studies^12^, we next tested whether increased VEGF-C might worsen metabolic parameters. To study the effect of VEGF-C overexpression, we utilized K14-VEGF-C transgenic mice^16^, which express human VEGF-C (hVEGF-C) under control of the keratin-14 promoter in epidermal keratinocytes. We found an expanded dermal lymphatic vascular network in the skin of K14-VEGF-C transgenic mice, reflecting efficient transgene expression (Fig. 1b). Preferential transgene production in the skin was confirmed by the detection of transgenic hVEGF-C in the SWAT (25.4 pg hVEGF-C/mg protein), that is in close proximity to the skin, of K14-VEGF-C mice, whereas serum hVEGF-C levels were below detection limit, potentially due to the short half-life (ca. 5 minutes) of VEGF-C in blood^17^ (Supplementary Fig. S1). Lymphatic vessels become largely independent of VEGF-C/VEGFR-3 signaling after the second week of postnatal life^18^,^19^ with the exception of intestinal (lymphatic) lacteals that depend on VEGF-C/VEGFR-3 signaling for their maintenance in adult mice^20^. To investigate whether overexpression of hVEGF-C in the skin altered the morphology or function of the intestinal lacteals, we performed whole-mount immunostainings of intestines together with an intestinal lipid uptake assay. Whole-mount immunostainings for LYVE-1 and CD31 revealed a comparable morphology of the lacteals in K14-VEGF-C and WT mice (Fig. 1b). The lipid uptake from the intestine was unaffected in K14-VEGF-C mice (Supplementary Fig. S1).

### K14-VEGF-C mice have increased weight gain and subcutaneous adipose tissue accumulation

The body weights of K14-VEGF-C mice and their WT littermate controls were monitored from 4 to 20 weeks of age. During this period, K14-VEGF-C transgenic mice gained significantly more weight than their WT littermates under chow diet (weight gain at week 20, WT: 12 ± 1.4 g, *n* = 8; K14-VEGF-C: 14.4 ± 2.5 g, *n* = 7; *p* = 0.033 with a two-tailed Student’s *t*-test; effect of genotype *p* < 0.0001 with a two-way ANOVA; Fig. 1c). Importantly, the mice had comparable weights at the start of the measurements. K14-VEGF-C transgenic mice also had a significantly increased % of SWAT (WT: 0.93 ± 0.49%, *n* = 8; K14-VEGF-C: 1.74 ± 0.49%, *n* = 7; *p* = 0.0075) and a trend towards an increased percentage of epididymal white adipose tissue (EWAT) (WT: 1.94 ± 0.63%, *n* = 8; K14-VEGF-C: 2.64 ± 0.84%, *n* = 7; *p* = 0.0863; Fig. 1d), indicating that the increased weight gain was at least partially due to an expansion of adipose tissue.

The transgenic hVEGF-C was detectable in SWAT but not in the serum of K14-VEGF-C mice. Accordingly, adipocytes in SWAT, but not EWAT (that is distal to the source of VEGF-C production) of K14-VEGF-C mice were significantly larger as compared to WT mice (median adipocyte size in SWAT: WT: 671.5 ± 108.3 μm^2^, *n* = 4; K14-VEGF-C: 1520 ± 395.2 μm^2^, *n* = 5; *p* = 0.0045; EWAT: WT: 2746 ± 680 μm^2^, *n* = 8; K14-VEGF-C: 2867 ± 790 μm^2^, *n* = 6; *p* = 0.7643; Fig. 1e,f). These results, in combination with the lack of apparent effects in the internal organs, indicated that the transgenic overexpression of hVEGF-C exerted its effects mainly on the tissues in close proximity to the source.

The proteolytically fully processed form of VEGF-C is able to bind and activate VEGFR-2^21^, and high levels of virally delivered VEGF-C have been reported to induce blood vessel sprouting and enlargement^22^. For this reason, we next analyzed the blood vessel morphology in skin sections by immunofluorescence stains for the blood vessel marker MECA-32. The average tissue area covered by blood vessels and the average blood vessel size were comparable in WT and K14-VEGF-C mice (Supplementary Fig. S2), in line with the initial reports that K14-VEGF-C mice showed no blood vessel angiogenesis^16^. Since hVEGF-C is produced in the skin and there was no angiogenesis of blood vessel at this location, we concluded that the hVEGF-C levels would be too low to induce angiogenesis elsewhere in the body.

### K14-VEGF-C mice develop insulin resistance

**Figure 2:**
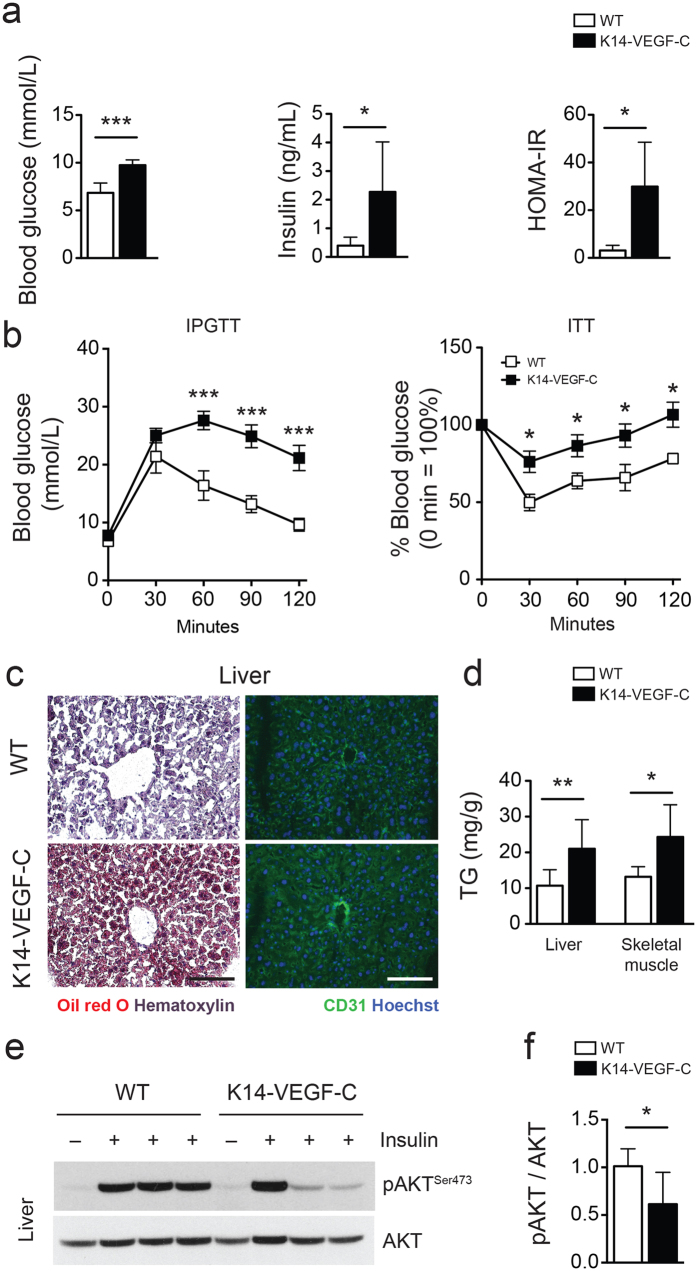
Reduced insulin sensitivity in K14-VEGF-C mice. (**a**) Elevated fasting blood glucose and insulin levels in K14-VEGF-C mice, leading to higher homeostatic model assessment of insulin resistance (HOMA-IR) indices. (**b**) Intraperitoneal glucose tolerance test (*n* = 4–6 per group, mean ± SEM, ***p < 0.001 compared to WT control at the same time point with a two-tailed Student’s *t*-test) and insulin tolerance test (*n* = 7 per group, mean ± SEM, *p < 0.05 compared to WT control at the same time point with a two-tailed Student’s *t*-test). (**c**) Oil Red O and CD31 staining of liver sections (scale bars = 100 μm). (**d**) Quantification of triglycerides (TG) in liver and skeletal muscle tissues. Representative western blots (**e**) and corresponding quantification (**f**) of phosho-AKT and AKT in liver lysates of WT and K14-VEGF-C mice. *p < 0.05, **p < 0.01 and ***p < 0.001, two-tailed Student’s *t*-test. Data are mean ± SD.

At 20 weeks of age, K14-VEGF-C mice had significantly elevated fasting blood glucose (WT: 6.9 ± 1.0 mmol/L, *n* = 8; K14-VEGF-C: 9.7 ± 0.5 mmol/L, *n* = 7; *p* < 0.0001) and fasting insulin levels (WT: 0.40 ± 0.29 ng/mL, *n* = 8; K14-VEGF-C: 2.28 ± 1.75 ng/mL, *n* = 7; Mann-Whitney *U* test *p* = 0.0426), which resulted in higher HOMA-IR indices (WT: 3.1 ± 2.1, *n* = 8; K14-VEGF-C: 25.8 ± 20.1, *n* = 7; Mann-Whitney *U* test *p* = 0.0111; Fig. 2a). The intraperitoneal glucose tolerance test (IPGTT) and insulin tolerance test (ITT) revealed marked impairment in glucose tolerance and response to insulin in K14-VEGF-C mice (Fig. 2b). In line with reduced insulin sensitivity, Oil Red O stainings of frozen liver sections revealed increased ectopic lipid accumulation in K14-VEGF-C mice (Fig. 2c). Accordingly, the triglyceride content of the livers (WT: 10.7 ± 4.5 mg/g tissue, *n* = 8; K14-VEGF-C: 21.0 ± 8.1 mg/g tissue, *n* = 6; Mann-Whitney *U* test *p* = 0.0047) and of the skeletal muscle (WT: 13.2 ± 2.8 mg/g tissue, *n* = 8; K14-VEGF-C: 24.4 ± 9.0 mg/g tissue, *n* = 7; Mann-Whitney *U* test *p* = 0.0159; Fig. 2d) was significantly elevated in K14-VEGF-C transgenic mice, confirming an increase in ectopic lipid accumulation.

As sinusoidal endothelia of the liver express VEGFR-3^23^, we investigated potential effects of transgenic VEGF-C overexpression on the liver vasculature. The blood vessel (visualized by CD31 staining) and the sinusoidal morphology of the livers were comparable in WT and K14-VEGF-C mice (Fig. 2c). Consistent with the insulin tolerance test and increased lipid accumulation, K14-VEGF-C mice also had a significantly blunted response to insulin stimulation, as shown by reduced AKT phosphorylation in the liver (Fig. 2e), whereas the livers of WT mice retained higher levels of phospho-AKT (pAKT/AKT ratio WT: 1.0 ± 0.2, *n* = 6; K14-VEGF-C: 0.6 ± 0.3, *n* = 7; *p* = 0.0251; Fig. 2f). Taken together, these data indicate that K14-VEGF-C mice developed insulin resistance under chow diet.

**Figure 3:**
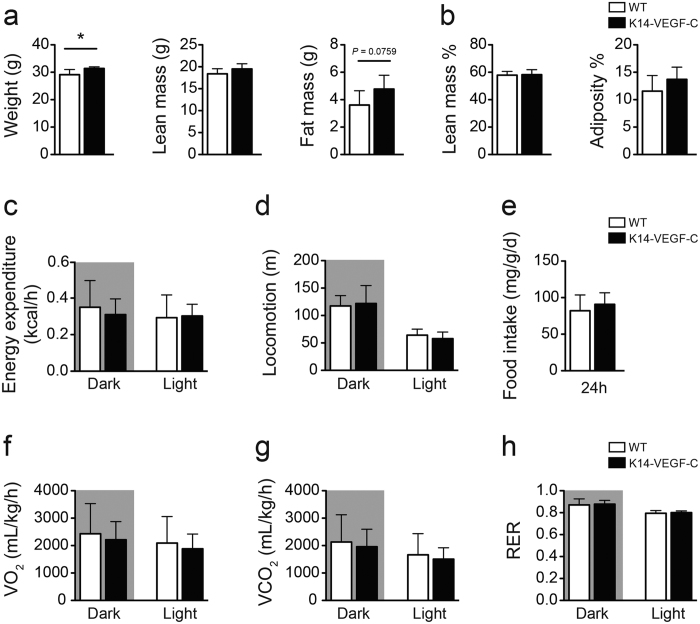
Energy balance parameters of K14-VEGF-C mice are similar to WT controls. (**a**) Body weight, lean mass and fat mass, (**b**) Lean mass % and adiposity %. (**c**) Energy expenditure (normalized to body weight and analyzed with ANCOVA). (**d**) Activity, (**e**) food consumption, (**f**) VO_2_, (**g**) VCO_2_ and (H) respiratory exchange rate (RER) were analyzed with indirect calorimetry using the Promethion system (*n* = 6 per group). *p < 0.05, two-tailed Student’s *t*-test. All data are mean ± SD.

To further investigate the metabolic phenotype of K14-VEGF-C mice, indirect calorimetry was performed using Promethion cages (Sable Systems International) in the Vanderbilt Mouse Metabolic Phenotyping Center. Body weight measurements and body composition analyses confirmed increased weight gain and elevated fat mass (WT: 3.6 ± 1.0 g, *n* = 6; K14-VEGF-C: 4.8 ± 1.0 g, *n* = 6; *p* = 0.0759) without changes in lean mass in K14-VEGF-C mice (Fig. 3a,b). Under ad libitum-fed conditions, the energy expenditure, total food intake and locomotion were similar (Fig. 3c–e). No differences were observed in total VO_2_, VCO_2_, or respiratory exchange rate (RER) between K14-VEGF-C and WT mice when normalized to body weight (Fig. 3f,g). Bomb calorimetry analysis of fecal samples showed no differences in the stool calorie content (Supplementary Fig. S3), suggesting that the observed phenotype was not caused by major differences in the energy intake/expenditure balance.

### K14-VEGF-C mice show metabolic deterioration also under HFD

**Figure 4:**
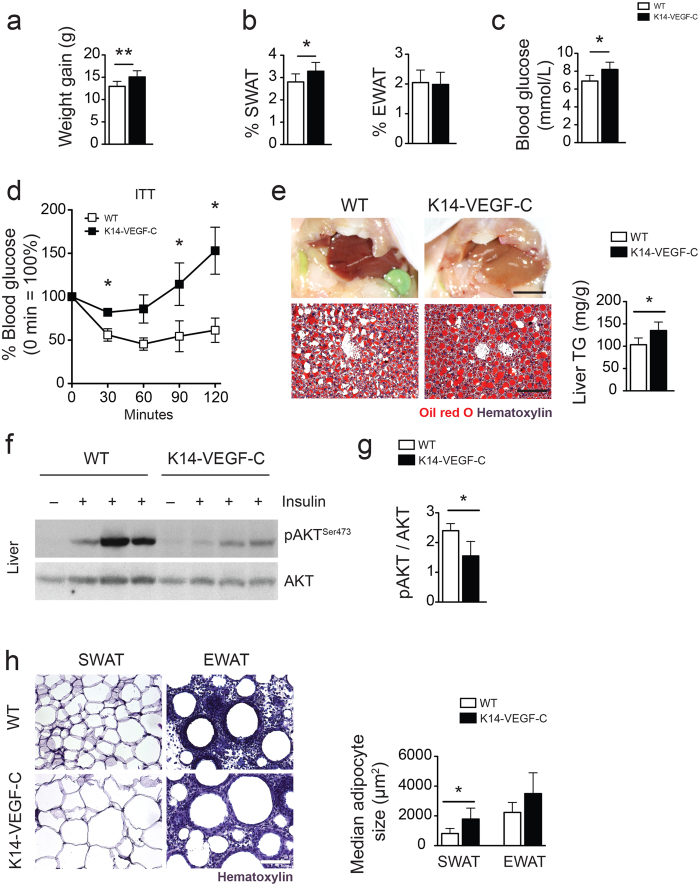
K14-VEGF-C mice show increased insulin resistance under HFD. (**a**) Increased body weight and (**b**) SWAT %, while EWAT % remained unaltered in K14-VEGF-C mice under HFD. (**c**) Fasting blood glucose was significantly elevated in K14-VEGF-C mice. (**d**) Insulin tolerance test (*n* = 5 per group, mean ± SEM is shown, *p < 0.05 compared to WT control at the same time point with a two-tailed Student’s *t*-test). (**e**) Comparison of liver gross morphology, Oil Red O stained liver sections and quantification of triglyceride (TG) content of livers showing increased ectopic lipid accumulation in K14-VEGF-C mice on HFD. Representative western blots (**f**) and corresponding quantification (**g**) of phosho-AKT and AKT in liver lysates of WT and K14-VEGF-C mice. (**h**) Cross sections of adipose tissue samples showing that K14-VEGF-C mice on HFD had larger SWAT adipocytes. Liver gross morphology images scale bar = 1 cm, liver Oil Red O staining and adipose tissue cross-section scale bars = 100 μm. *p < 0.05 and **p < 0.01, two-tailed Student’s *t*-test. Data are mean ± SD.

To study the effects of diet-induced obesity, 4-week-old K14-VEGF-C mice and their WT littermates were fed with a HFD for 12 weeks. K14-VEGF-C mice under HFD showed significantly increased weight gain (WT: 13.0 ± 0.4 g, *n* = 7; K14-VEGF-C: 15.1 ± 0.5 g, *n* = 7; *p* = 0.0089; Fig. 4a), and SWAT %, but not EWAT % (Fig. 4b). K14-VEGF-C mice also had higher fasting blood glucose levels than WT mice (WT: 6.9 ± 0.3 mmol/L, *n* = 6; K14-VEGF-C: 8.2 ± 0.3 mmol/L, *n* = 6; *p* = 0.0114; Fig. 4c). Similar to the findings under chow diet, K14-VEGF-C mice under HFD had significantly larger adipocytes in the SWAT than WT mice under the same diet (median adipocyte size in SWAT: WT: 817 ± 338 μm^2^, *n* = 5; K14-VEGF-C: 1784 ± 738 μm^2^, *n* = 6; *p* = 0.0045). The insulin tolerance test revealed a significant decrease in the response to insulin in K14-VEGF-C mice (Fig. 4d). The livers of K14-VEGF-C mice contained higher amounts of lipids and as a result were more yellow (Fig. 4e). Oil Red O staining of frozen liver sections revealed increased ectopic lipid accumulation in K14-VEGF-C mice (Fig. 4e). These livers contained significantly higher levels of triglycerides (WT: 10.7 ± 4.5 mg TG/g liver, *n* = 8; K14-VEGF-C: 21.0 ± 8.1 mg TG/g liver, *n* = 6; *p* = 0.0099; Fig. 4e), confirming increased ectopic lipid accumulation in K14-VEGF-C transgenic mice under HFD. In line with this, K14-VEGF-C mice under HFD showed an almost complete abolishment of AKT phosphorylation in the liver upon insulin stimulation (Fig. 4f), whereas the livers of WT mice maintained higher levels of AKT phosphorylation, suggesting a profound decrease in insulin sensitivity in K14-VEGF-C transgenic mice (pAKT/AKT ratio WT: 2.4 ± 0.2, *n* = 4; K14-VEGF-C: 1.6 ± 0.5, *n* = 3; *p* = 0.0271; Fig. 4g).

### Enhanced pro-inflammatory phenotype of K14-VEGF-C SWAT macrophages

**Figure 5:**
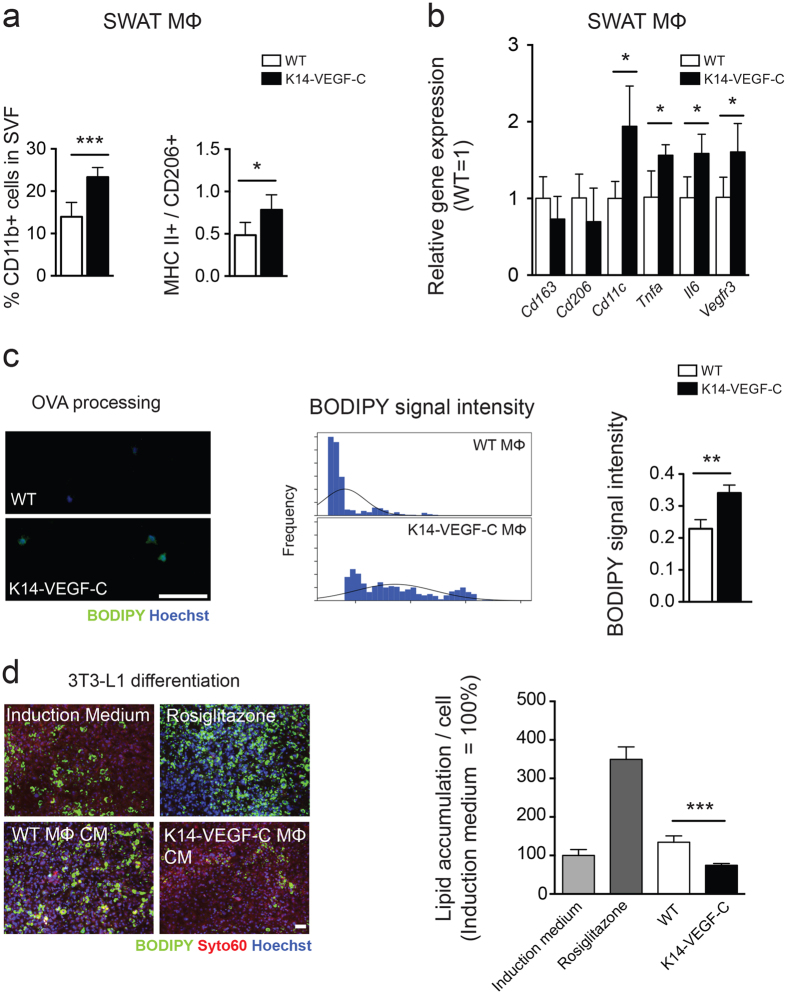
SWAT macrophages of K14-VEGF-C mice show enhanced pro-inflammatory characteristics before the onset of increased adiposity. (**a**) Data from 22-week-old mice, (**b–d**) data from 14-week-old mice; prior to the onset of weight gain. (**a**) Increased percentage of CD11b+ cells in SWAT stromal vascular fraction, with a significant elevation of M1/M2 macrophage marker ratios in K14-VEGF-C mice. (**b**) Gene expression analysis of M2 (CD163, CD206) and M1 (CD11c, TNF-α, IL6) markers showed a boosted M1 phenotype in isolated K14-VEGF-C SWAT macrophages from 14-week-old mice (*n* = 3–4). (**c**) Increased BODIPY fluorescence signal revealed by enhanced OVA processing of K14-VEGF-C SWAT macrophages; histograms show the distribution of BODIPY signal intensity and the corresponding quantification per mouse (*n* = 3 mice per group, scale bar = 100 μm). (**d**) Conditioned media from SWAT macrophages of K14-VEGF-C, but not WT mice, significantly reduced the differentiation of 3T3-L1 cells *in vitro* (*n* = 4 mice per group, scale bar = 200 μm). **p < 0.01, ***p < 0.001, two-tailed Student’s *t*-test. Data are mean ± SD.

We have previously demonstrated that blockade of VEGF-C/-D in SWAT results in enhanced anti-inflammatory (M2 polarized) macrophage accumulation and an increased M2/M1 ratio^12^. When IFN-γ and LPS treatments were used to polarize macrophages into a pro-inflammatory phenotype (M1 polarized), the macrophages were shown to upregulate VEGFR-3^12^,^14^. As VEGF-C induces chemotaxis of macrophages via VEGFR-3 signaling, we hypothesized that overexpression of VEGF-C in SWAT would result in an increase of M1 macrophages. To address if this is the case, we performed flow cytometry analyses of 22-week-old mice and found that K14-VEGF-C SWAT contains significantly more CD11b+ cells in the stromal vascular fraction (WT: 14.0 ± 3.4%, *n* = 6; K14-VEGF-C: 23.3 ± 2.3, *n* = 6; *p* = 0.0002; Fig. 5a) and a significantly higher M1/M2 macrophage ratio –as determined by the ratio of MHC II + % (M1 marker) to CD206 + % (M2 marker) cells– within the F4/80/CD11b double positive macrophage populations (WT: 0.48 ± 0.15, *n* = 6; K14-VEGF-C: 0.78 ± 0.18, *n* = 6; *p* = 0.0101; Fig. 5a). In order to investigate whether the macrophages in the SWAT have a pro-inflammatory phenotype prior to the development of weight gain and insulin resistance, we used 14-week-old female mice (which do not have increased weight gain at this age unlike the male mice) and characterized the polarization characteristics of the SWAT macrophages. Gene expression analyses showed that while the M2-associated genes *Cd163* and *Cd206* were downregulated, the M1-associated genes *Cd11c*, *Tnfa* and *Il6* were significantly upregulated in K14-VEGF-C SWAT macrophages (Fig. 5b). Interestingly, and as expected, SWAT macrophages of K14-VEGF-C mice had significantly higher *Vegfr3* mRNA levels (Fig. 5b).

A cardinal property of pro-inflammatory M1 macrophages is their superior antigen processing and presenting capacity as compared to M2 macrophages. In order to measure the antigen processing potential of the SWAT macrophages, we isolated CD11b+ macrophages from subcutaneous adipose tissue of 14-week-old female mice and incubated them with a self-quenching Ovalbumin-BODIPY (OVA-BODIPY) conjugate. This conjugate is taken up by antigen presenting cells and only after the OVA peptide is processed, an increase in BODIPY signal can be detected. After 2 hours of incubation, the macrophages from the SWAT of K14-VEGF-C transgenic mice had an approximately 1.5-fold higher average fluorescence intensity than the wildtype macrophages (Fig. 5c). When the individual cell signal intensity data were plotted as histograms, the signal intensity peak of K14-VEGF-C SWAT macrophages had a shift towards right, indicating an increased number of macrophages with higher BODIPY fluorescence (Fig. 5c).

We next investigated if hVEGF-C can exert its effects directly by modulating adipogenic differentiation. To do so, we performed *in vitro* adipogenic differentiation assays with 3T3-L1 cells and stromal-vascular fractions from murine SWAT. We found that while hVEGF-C did not affect adipogenic differentiation and lipid accumulation of 3T3-L1 cells (cell line), it did enhance the adipogenic differentiation of stromal-vascular fractions (primary cells), which consist of a mixed cell population (Supplementary Fig. S4). In order to investigate whether the effects seen in the SWAT, but not in the EWAT, were due to differences in *Vegfr3* expression, we next analyzed *Vegfr3* gene expression levels. Our findings indicate that under steady state conditions, the expression of *Vegfr3* in these two adipose tissue depots is similar (Supplementary Fig. S5).

As pro-inflammatory cytokines have been reported to block the adipogenic differentiation of 3T3-L1 cells *in vitro*^24^, we investigated the effects of conditioned media of the SWAT macrophages. As expected, and in line with gene expression and antigen processing data, the conditioned media of K14-VEGF-C, but not of wildtype SWAT macrophages significantly blocked adipogenic differentiation of 3T3-L1 cells. Together, these data strongly suggest that inflammatory macrophages infiltrate K14-VEGF-C SWAT prior to weight gain and metabolic deterioration.

## Discussion

In this study, we investigated the impact of excess VEGF-C on metabolism in mice under normal chow and HFD conditions. Our findings reveal that transgenic overexpression of VEGF-C in mice induces a moderate yet significant weight gain, adipocyte hypertrophy, ectopic lipid accumulation in the liver and insulin resistance under both diets via recruitment of inflammatory macrophages into adipose tissue.

In our previous study, VEGF-C and VEGF-D blockage protected the mice against obesity-induced insulin resistance and hepatic steatosis by favoring a higher anti-inflammatory/pro-inflammatory macrophage ratio in SWAT, which supported adipocyte differentiation^12^. Mechanistic studies revealed that VEGF-C and -D preferentially recruit pro-inflammatory macrophages that express their cognate receptor VEGFR-3^12^,^14^. Thus, we hypothesized that overexpression of VEGF-C in our transgenic model might recruit pro-inflammatory macrophages into SWAT, which would result in an increase of inflammatory cytokines that can block adipogenic differentiation and cause adipocyte hypertrophy and insulin resistance^24^,^25^,^26^. Indeed, our data reveal that inflammatory macrophages infiltrate the SWAT of K14-VEGF-C mice prior to the emergence of weight gain and insulin resistance, despite the fact that VEGF-C can induce adipogenic differentiation of stromal-vascular fractions. Based on our data we propose that the observed effects on metabolic control and adipocyte size are not due to the cell autonomous induction of adipogenesis but rather due to the generation of a pro-inflammatory environment that causes downstream detrimental metabolic effects.

Analyses of previously published datasets show a correlation of *Vegfc* expression and diet and caloric intake in metabolically active tissues. For instance, *Vegfc* mRNA in the liver was significantly upregulated upon HFD in 12 different inbred mouse strains^27^,^28^. Increased *Vegfc* levels were also reported in the EWAT of mice fed a HFD (GEO Accession GSE 63198). On the other hand, a 40% caloric restriction reduced *Vegfc* gene expression levels in gastrocnemius muscles^29^ and EWAT of C57BL/6 J mice^30^. *VEGFC* mRNA levels in human skeletal muscle were 2.5 fold lower in people subjected to 30% caloric restriction than in humans on Western diet^31^, suggesting a link between diet and VEGF-C levels in metabolically active tissues.

As elevated VEGF-C levels might have a systemic effect, we measured serum concentrations of hVEGF-C in K14-VEGF-C mice and found that the hVEGF-C levels were below detection limit of the used kit (48.4 pg/mL serum). Since (1) serum levels of endogenous (mouse) VEGF-C have been reported to be around 50–100 pg/mL^14^, (2) mouse and human VEGF-C have similar receptor binding affinities, (3) the lowest detectable amount of hVEGF-C in serum is lower than endogenous VEGF-C levels, and (4) the hVEGF-C levels detected in SWAT are 50 fold higher than the endogenous VEGF-C levels, we think that in our model a local effect of the transgenically produced hVEGF-C is more likely.

The K14-VEGF-C mice weighed about 2.5 g more than WT mice at 20 weeks of age under chow diet. When the mice were kept on HFD for 12 weeks, the difference in weight gain was maintained. Interestingly, despite a moderately increased weight gain, there was a marked effect on insulin sensitivity. The hypertrophic subcutaneous adipocytes observed in K14-VEGF-C mice provide a potential explanation for this. Although the contribution of adipose tissue to glucose clearance is estimated to be only about 10%^32^, the insulin sensitive adipocytes may play a major role in buffering lipids and protecting other organs from ectopic fat accumulation^33^. The adipocyte hypertrophy seen in K14-VEGF-C mice could lead to a reduction in adipocyte insulin sensitivity, which in turn could promote ectopic lipid accumulation in the liver and evoke insulin resistance in this model.

Interestingly, despite the differences in the insulin sensitivity, we did not observe obvious differences in the energy balance parameters during the observation period. This might be due to several reasons. First, single housing may have altered the energy metabolism or metabolic rate of the mice, as the mice lost approximately 1.5 g of body weight during indirect calorimetry measurements. Indirect calorimetry experiments likely stress the mice and therefore might have induced the observed weight loss. Similarly, small differences in food intake and energy extraction in the gut, which were not observed during the metabolic cage assessments or with fecal calorie content analyses, may have contributed to weight gain over time. Second, weight gain without changes in energy balance parameters could stem from differences in gut microbiota profiles. The gut microbiota is an integral component of the digestive system that facilitates energy extraction from food and contributes to the production of a wide variety of metabolites and growth factors^34^. Interestingly, changes in gut microbiota can alter the caloric yield of diet, thereby affecting energy metabolism^35^,^36^. Gut microbiota was shown to mediate energy storage in adipocytes by suppressing the intestinal production of fasting-induced adipocyte factor (Fiaf), a lipoprotein lipase inhibitor^37^. Since VEGF-C/VEGFR-3 signaling has been implicated in the modulation of experimental inflammatory bowel disease in mice^38^,^39^, it is possible that increased VEGF-C levels might alter the gut immune system and the profile of the microbiota, which in turn might promote weight gain and insulin resistance in the K14-VEGF-C mice.

In conclusion, the results from this study, taken together with our previous results showing an improvement of metabolic parameters when VEGF-C/VEGFR-3 signaling was blocked in obesity, suggest that VEGF-C contributes to the development of insulin resistance and that neutralization of VEGF-C during obesity represents a new and promising strategy to improve insulin sensitivity in the metabolic syndrome.

## Methods

### Mice

Male K14-VEGF-C transgenic mice on the FVB background, that express human VEGF-C under control of the keratin 14 promoter^16^, and their wildtype littermates were kept under specific pathogen free (SPF) conditions. Starting at the age of 4 weeks, the mice were kept *ad libitum* either on a control diet (chow; 11% kcal from fat, 31% kcal from protein and 58% kcal from carbohydrate; Provimi-Kliba, Kaiseraugst, Switzerland) for 16–18 weeks, or a high-fat diet (HFD, 60% kcal from lard (fat from pig), 20% kcal from protein and 20% kcal from carbohydrate; Research Diets Inc., NJ, USA) for 12 weeks. Mice were weighed every two weeks. All experiments were performed in accordance with animal protocols approved by the Kantonales Veterinäramt Zürich.

### ELISA

Subcutaneous white adipose tissue (SWAT) and serum samples were collected and snap-frozen. Proteins were extracted from the SWAT samples using a modified RIPA buffer as described^12^. Mouse VEGF-C levels were measured using a mouse VEGF-C ELISA kit (Cusabio) and human VEGF-C (hVEGF-C) levels were measured using a human Quantikine ELISA kit for VEGF-C (R&D) following the manufacturers’ instructions.

### Histology, immunofluorescence and image acquisition

PFA-fixed paraffin embedded SWAT and epididymal white adipose tissue (EWAT) samples were sectioned (10 μm) and incubated overnight at 65 °C. The sections were deparaffinized, rehydrated and stained with hematoxylin. Ten images per section were acquired at 20X magnification and adipocyte size was measured using CellProfiler software ( http://www.cellprofiler.org). Tail or liver cryosections (7 μm) were fixed with acetone (−20 °C) and 80% methanol (4 °C), washed in PBS and then incubated overnight with a hamster anti-podoplanin antibody (clone 8.1.1, Developmental Studies Hybridoma Bank, University of Iowa) and a rat MECA-32 antibody (1:200, BD Pharmingen) for visualizing lymphatic and blood vessels in tail sections, and rat anti-CD31 antibody (1:150, BD Pharmingen) to visualize blood vessels in liver sections, respectively. Alexa488- and Alexa594-conjugated secondary antibodies (1:200) and Hoechst 33342 (1:1000) were purchased from Invitrogen (Invitrogen, Basel, Switzerland), and images were acquired at 20X magnification. For bright field and fluorescence imaging, an Axioskop 2 mot plus microscope (Carl Zeiss, Inc.), equipped with a Plan-APOCHROMAT 10×/0.45 NA objective, an AxioCam MRc camera and a Plan-NEOFLUAR 20×/0.50 objective (both from Carl Zeiss) were used. For imaging of 3T3-L1 cell cultures, a Zeiss Axiovert 200 M microscope equipped with a Zeiss AxioCam MRm camera with maximum contrast, equipped with an LD-Plan NEOFLUAR 20×/0.4 PhD2 Korr objective (Carl Zeiss) were used, and the images were acquired with Axiovision software (version 4.7.1). Confocal imaging was performed on a Zeiss LSM 710-FCS confocal microscope equipped with a 10 × 0.3NA EC Plan-Neofluar objective and a 20 × 0.8 NA Plan-Apochromat objective (all from Carl Zeiss). Images were acquired using the Zeiss ZEN 2009 software and processed using Imaris software (version 7.5.1, Bitplane) or ImageJ (NIH).

### Intestinal lipid uptake

Lipid uptake was studied as described^12^, using 8-week-old male K14-VEGF-C mice or WT littermates.

### Whole-mount immunofluorescence stains

Whole-mount immunostainings were performed as described^12^. For details see Supplementary Information.

### Fat pad weights, body composition analyses and indirect calorimetry

Posterior subcutaneous fat pads and epididymal fat pads were excised, weighed and expressed as % body weight. Body composition analyses and indirect calorimetry were performed using Promethion cages (Sable Systems International) at the Vanderbilt Mouse Metabolic Phenotyping Center by monitoring 30-week-old K14-VEGF-C and WT controls for 2 weeks in single housed cages.

### Fasting glucose and insulin measurements

Male K14-VEGF-C mice and WT littermate controls that were under chow diet for 15 weeks (*n* = 7 each) or under HFD for 11 weeks (*n* = 5 each) were fasted for 8 h (6 AM to 2 PM, during light cycle) and fasting blood was collected for glucose and insulin measurements. Fasting glucose was measured with a Contour glucometer (Bayer HealthCare) and fasting insulin was measured in serum using a high-sensitivity mouse insulin ELISA kit (Crystal Biochem).

### Insulin and glucose tolerance tests

For the insulin tolerance test, male K14-VEGF-C mice and WT littermate controls that were under chow diet for 15 weeks (*n* = 7 each) or under HFD for 11 weeks (*n* = 5 each) were fasted for 8 h (6 AM to 2 PM, during light cycle) and fasting blood glucose was measured as described above. The mice were then injected with 0.75U (chow) or 2U (HFD) of insulin intraperitoneally. The homeostatic model assessment of insulin resistance (HOMA-IR) index was calculated using glucose and insulin concentrations of fasting blood, using the following formula: fasting blood glucose (mmol/L)× fasting insulin (μU/mL)/22.5. For the glucose tolerance tests, male mice that were under chow diet for 17 weeks (*n* = 4–6 per group) were fasted overnight and fasting glucose was measured. Thereafter, the mice were injected intraperitoneally with 2 g/kg glucose and the blood glucose was monitored over 2 h.

### Insulin stimulated AKT phosphorylation

Insulin stimulated AKT phosphorylation experiments and following western blots were performed with male K14-VEGF-C mice and WT littermate controls that were under chow diet for 15 weeks or under HFD for 11 weeks as described^12^.

### Tissue triglyceride quantification

The triglycerides in the liver and skeletal muscle were extracted as previously described^12^ from 50 mg of tissue and were measured with the BioVision kit following the manufacturer’s instructions.

### Flow cytometry

Flow cytometry of SWAT macrophages was performed using 22-week-old male mice as previously described^12^, using the following antibodies: F4/80-APC and CD11b-FITC (both from eBioscience, 1:100) to gate double-positive cells, and CD206-PE and MHCII-PerCP (both from Biolegend, 1:100) to assess distinct macrophage subsets.

### Adipogenic differentiation of 3T3-L1 cells and SVF

Adipogenic differentiation experiments were performed as described^12^,^40^,^41^. For details see Supplementary Information.

### Conditioned media collection

Conditioned media were collected as described previously^12^. Briefly, MACS-isolated macrophages from 14-week-old mice were seeded in 12-well plates (50.000 cells/well) in DMEM (Gibco), supplemented with 1% FBS. After 24 h, the supernatant was collected, centrifuged and stored at −20 °C until used in 3T3-L1 differentiation assays.

### Ovalbumin processing

After overnight incubation of macrophages in 8-well-chambered glasses, 5 μL (1:100 dilution) of BODIPY-conjugated Ovalbumin peptide (DQ Ovalbumin, Invitrogen, 1 mg/mL in PBS) was added into each well and the CD11b+ cells were allowed to process the DQ Ovalbumin for 2 hours at 37 °C in a humidified incubator. The cells were fixed with 100% methanol for 5 minutes at 4 °C and were briefly washed with PBS. Thereafter, the cells were counterstained with Hoechst and imaged. Approximately 5 images per mouse were acquired with a fluorescence microscope (Carl Zeiss) at 20X magnification and the average intensities of BODIPY signals per cell in each image were measured with CellProfiler Software.

### Statistical analyses

Data are represented as mean ± SD unless otherwise stated in the figure legends. The weight-gain data were analyzed with repeated measures ANOVA and single time points were compared with the two-tailed Student’s *t*-test. All data were tested for normality using a Shapiro-Wilk test. Means of two groups were compared with the two-tailed Student’s *t*-test, a Welch correction was used in case of unequal variances.

A Mann-Whitney *U* test was used to compare non-normally distributed data. Energy expenditure data were analyzed using ANCOVA (normalized to body weight) in accordance with current guidelines^42^. We performed the analyses and plotted the graphs using GraphPad Prism V5.0 for MacOSX (GraphPad Software, San Diego California, USA) and SPSS Statistics 22.0 (SPSS Inc, Chicago, IL). A *p* value less than 0.05 was accepted as statistically significant.

## Additional Information

**How to cite this article**: Karaman, S. *et al.* Transgenic overexpression of VEGF-C induces weight gain and insulin resistance in mice. *Sci. Rep.* **6**, 31566; doi: 10.1038/srep31566 (2016).

## Supplementary Material

Supplementary Information
