# Supplementary material for: Transgenic overexpression of VEGF-C induces weight gain and insulin resistance in mice
Source: Sci Rep. 2016 Aug 11;6:31566. doi: 10.1038/srep31566 (PMC4980670; doi:10.1038/srep31566)
Supplement: Supplementary Information [file srep31566-s1.pdf]

SUPPLEMENTARY INFORMATION

Transgenic overexpression of VEGF-C induces weight gain and insulin resistance in mice

Sinem Karaman[1], Maija Hollmén[1], Sun-Young Yoon[1], H. Furkan Alkan[1], Kari Alitalo[2], Christian Wolfrum[3] and Michael Detmar[1]

[1]Institute of Pharmaceutical Sciences, Swiss Federal Institute of Technology, ETH Zurich, 8093 Zurich, Switzerland;

[2]Wihuri Research Institute and Translational Cancer Biology Program, Biomedicum Helsinki, University of Helsinki, Finland;

[3]Institute of Food, Nutrition and Health, Swiss Federal Institute of Technology, ETH Zurich, 8603 Schwerzenbach, Switzerland

SUPPLEMENTARY METHODS

Whole mount immunofluorescence stains

Briefly, 1 cm long pieces of small intestine were dissected and cut longitudinally, washed with PBS and fixed in 4% PFA for 1 h at 4ºC. The samples were then blocked with a mixture of 5% donkey serum, 0.1% Triton-X, 1% BSA and 0.05% sodium azide in PBS for 2 h, and then incubated with the primary antibodies overnight. This was followed by several washes and incubation with secondary antibodies for 2 h. Finally, samples were washed for 2 h with PBS and flat-mounted on glass slides with Mowiol. Z-stack images were acquired with a Zeiss LSM 710-FCS confocal microscope. Primary antibodies used were rabbit anti-mouse LYVE-1 (AngioBio, 1:600) and rat anti-mouse CD31 (BD Pharmingen, 1:250). Alexa 594- and 647-conjugated secondary antibodies were purchased from Invitrogen and used at a 1:200 dilution.

3T3-L1 adipogenic differentiation assays

Adipogenic differentiation assays were performed as previously described [1]. 3T3-L1 mouse embryonic fibroblasts (preadipocytes) were expanded in high-glucose (4.5 g/L) Dulbecco's modified Eagle's medium (DMEM; Gibco) supplemented with 10% bovine serum (Sigma) and penicillin/streptomycin. The cells were then plated onto collagen type I coated plates and were incubated in high-glucose DMEM, supplemented with 10% fetal bovine serum and penicillin/streptomycin (all from Gibco) in a humidified incubator at 5% CO_2. 48 hours post-confluence, adipogenic differentiation was induced with insulin (172 nM), dexamethasone (1 μM) and isobutyl-methylxanthine (115 μg/mL) (all from Sigma). To test if VEGF-C has an

effect on adipogenic differentiation of 3T3-L1 cells, 200 ng/mL human VEGF-C (kind gift from M. Jeltsch, University of Helsinki, Finland [2]) or an equivalent volume of PBS were added to the induction medium. Rosiglitazone (10 µM) was used as a positive control for the induction of adipogenic differentiation. After 2 days, the medium was changed to insulin medium, which contained only 172 nM insulin and 200 ng/mL VEGF-C or equivalent volume of PBS for the experimental conditions, which was applied every two days. For analyzing the effect of macrophage-secreted cytokines on adipogenic differentiation, the medium was supplemented with 10% macrophage conditioned medium using the same set up. At day 10, cells were washed twice with PBS, fixed with 4% PFA and stained with BODIPY 493/503, Syto60 and Hoechst (all from Invitrogen) to stain lipid droplets, cytosol and nuclei, respectively. The percentage of differentiated adipocytes was analyzed with CellProfiler software as previously described [3] and the adipogenic differentiation observed in induction medium was set to 100%.

SVF differentiation assay

SVF isolation, differentiation and quantification were performed as previously described [3, 4]. Briefly, SVF from mouse subcutaneous adipose tissue was isolated and plated on collagen-coated plates in 96-well format. The adipogenic differentiation was induced when cells reached 80% confluence and 200 ng/mL hVEGF-C (kind gift from M. Jeltsch, University of Helsinki, Finland [2]) or PBS in equivalent volume was applied on days 0, 2, 4 and 6 of differentiation. Thereafter, the cells were fixed and stained on day 8, and 9 images were acquired per well using an automated microscope system (CellWorks, Thermo Fisher). Images were analyzed as described above.

Bomb calorimetry of stool samples

Fecal energy content was measured by bomb calorimetry. Briefly, stool samples were collected from 17-week old mice that were on chow diet or HFD (n = 6-8 per group) and dried overnight at 37°C and the energy content of pooled samples from each genotype was analyzed by using an IKA Calorimeter (IKA-Werke GMBH).

Analysis of gene expression

RNA was isolated from SWAT and EWAT samples using RNeasy Mini Kit (Qiagen) and from SWAT macrophages using RNeasy Micro Kit (Qiagen) following manufacturer's instructions. cDNA was transcribed from 1 µg of RNA using the High Capacity Reverse Transcription kit (Applied Biosystems). The PCR reactions were performed using FastStart SYBR green master mix (Roche) and a 7900HT Fast Real-Time PCR System (Applied Biosystems) using the following primer sets [1], *Vegfr3*-Forward: 5′- CTGGCAAATGGTTACTCCATGA-3′, *Vegfr3*-Reverse: 5′-ACAACCCGTGTGTCTTCACTG-3′, and *Rplp0* as internal control *Rplp0*-Forward: 5′-AGATTCGGGATATGCTGTTGGC-3′, *Rplp0*-Reverse: 5′-TCGGGTCCTAGACCAGTGTTC-3′ and gene expression fold changes were calculated using the $\Delta\Delta_{CT}$ method.

SUPPLEMENTARY FIGURES and FIGURE LEGENDS

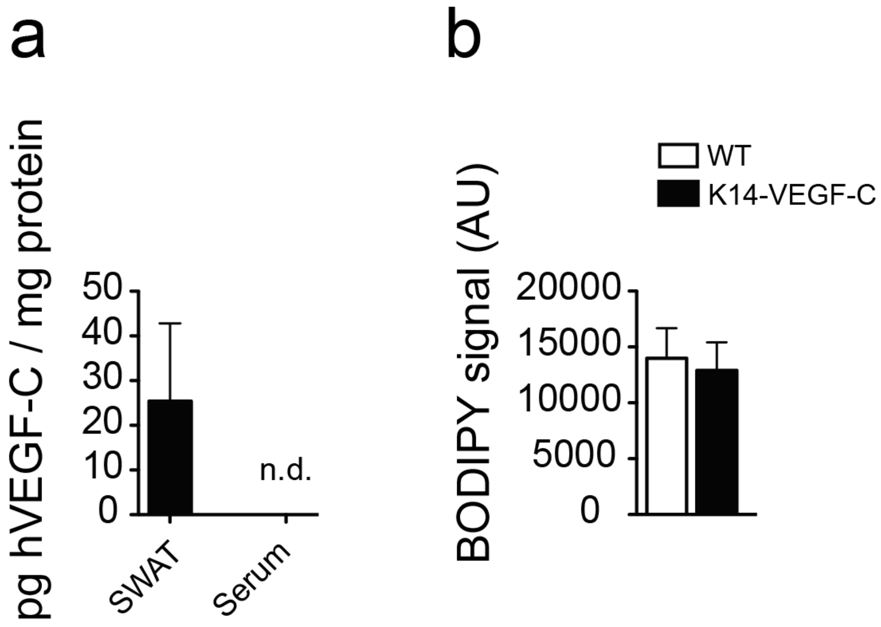

Supplementary Figure S1: Overexpressed hVEGF-C could be detected in SWAT and does not affect lipid uptake from intestinal lacteals. (**a**) ELISA analysis of SWAT and serum samples showing detectable amounts of hVEGF-C in SWAT (*n*=5 per group) but not in serum. Tissue samples from WT mice were used as controls as they do not contain hVEGF-C (not shown). n.d., not-detected. (**b**) BODIPY fluorescence measured in the serum 1 h after oral gavage revealed comparable lipid uptake in WT and K14-VEGF-C mice (*n*=3-4, two-tailed Student's *t*-test). Data are mean ± SD.

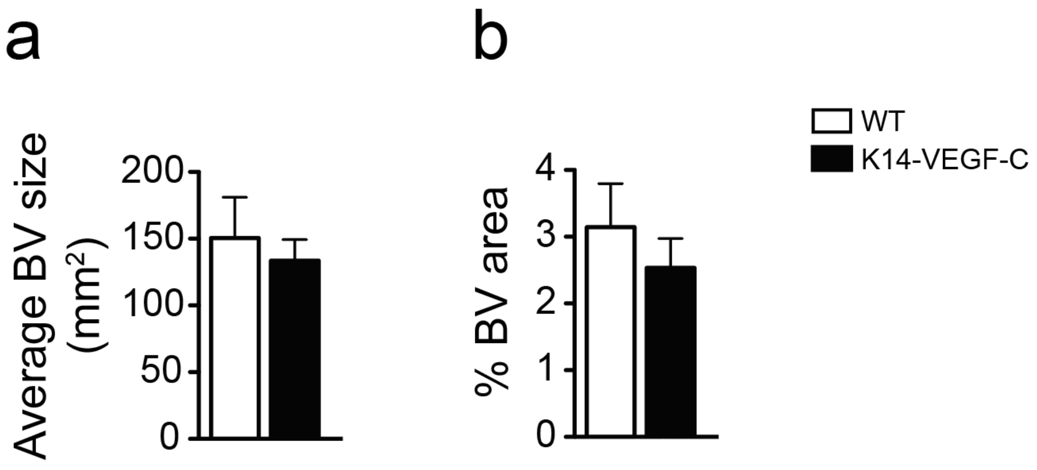

Supplementary Figure S2: Blood vasculature in the skin is not affected by transgenic overexpression of hVEGF-C. (**a**) Average blood vessel (BV) size and (**b**) % area covered by blood vessels (normalized to the entire region of interest) were measured from immunofluorescence stains of skin sections (shown in Figure 1B) of 20-week-old WT and K14-VEGF-C mice, revealing comparable blood vascularity at the site of transgenic overexpression (*n*=5 per group, two-tailed Student's *t*-test). Data are mean ± SD.

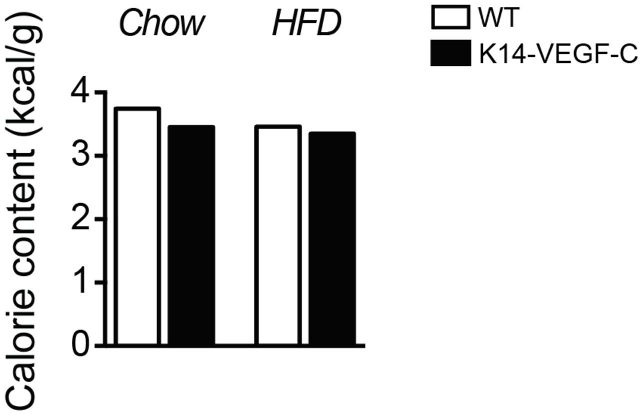

Supplementary Figure S3: Energy content of stool samples. Bomb calorimetry of fecal samples showed no major differences in the energy content. Data from pooled samples is shown (*n*=6 for WT groups and *n*=8 for K14-VEGF-C groups).

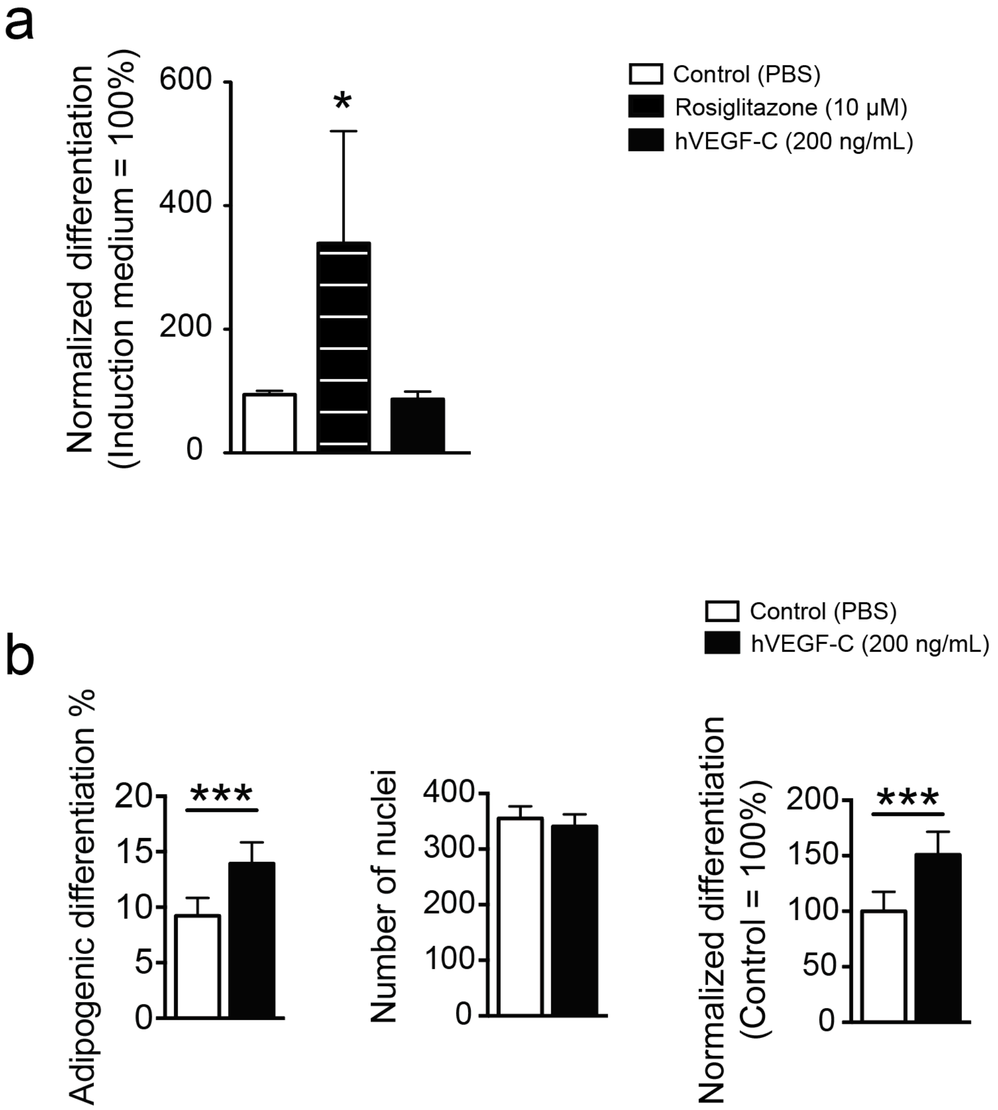

Supplementary Figure S4: Effect of VEGF-C on adipogenic differentiation. (**a**) No differences in adipogenic differentiation were detected when 3T3-L1 cells were treated with 200 ng/mL hVEGF-C, whereas the positive control Rosiglitazone (10 µM) promoted adipogenic differentiation. Results showing mean ± SD of three independent experiments. *$p<0.05$, one-way ANOVA followed by Dunnett's multiple comparison post-hoc test. (**b**) On the other hand, 200 ng/mL hVEGF-C application induced adipogenic differentiation of SWAT stromal vascular fractions as compared to control without affecting cell proliferation ($n=12$ per group, two-tailed Student's *t*-test). *$p<0.0001$. Data are mean ± SD.

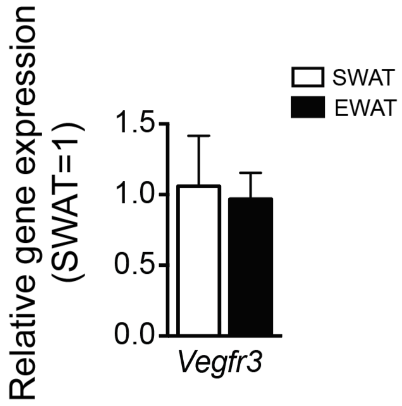

Supplementary Figure S5: *Vegfr3* gene expression in SWAT and EWAT. No differences in *Vegfr3* gene expression were found between SWAT and EWAT under steady state conditions (*n*=8 per group). Data are mean ± SD.

SUPPLEMENTARY REFERENCES

1. Karaman, S. *et al.* Blockade of VEGF-C and VEGF-D modulates adipose tissue inflammation and improves metabolic parameters under high-fat diet. *Mol Metab* **4**, 93–105 (2015).

2. Karpanen, T. *et al.* Lymphangiogenic growth factor responsiveness is modulated by postnatal lymphatic vessel maturation. *Am J Pathol* **169**, 708–718 (2006).

3. Meissburger, B. *et al.* Adipogenesis and insulin sensitivity in obesity are regulated by retinoid-related orphan receptor gamma. *EMBO Mol Med* **3**, 637–651 (2011).

4. Meissburger, B. *et al.* Tissue inhibitor of matrix metalloproteinase 1 (TIMP1) controls adipogenesis in obesity in mice and in humans. *Diabetologia* **54**, 1468-79 (2011).
